# Retrograde filling of an internal iliac artery aneurysm via the profunda femoris artery: A case report of type IIa endoleak

**DOI:** 10.1016/j.ijscr.2024.110682

**Published:** 2024-11-29

**Authors:** Abdullah G. Alsahwan, Ahmed Almumtin, Mohammed A. Sadig, Osama Alahmadi, Shagran Binkhamis, Omer Abdulrahim

**Affiliations:** a King Faisal Specialist Hospital and Research Center, Riyadh, Saudi Arabia; b Department of Surgery, King Fahad Hospital of the University, Imam Abdulrahman Bin Faisal University, Al-Khobar, Saudi Arabia; c Department of Surgery, Faculty of Medicine, University of Dongola, Dongola, Sudan

**Keywords:** Internal iliac artery aneurysm, Endoleak, Profunda femoris artery, Type IIa endoleak, Endovascular repair

## Abstract

**Introduction:**

Internal iliac artery aneurysms (IIAAs) are an uncommon but clinically significant vascular condition that can lead to life-threatening complications, such as rupture and endoleaks, following endovascular repair. Endoleaks particularly type IIa, occur when there is retrograde flow into the aneurysm sac from collateral vessels, and their presence can jeopardize the success of repair procedures. This case report illustrates a rare occurrence of a type IIa endoleak attributed to retrograde filling from the profunda femoris artery, providing insights into the diagnostic complexities and management of IIAAs.

**Case presentation:**

A 32-year-old male with a history of hypertension and end-stage renal disease, who was incidentally found to have a large, partially thrombosed IIAA while undergoing evaluation for kidney transplantation. The initial endovascular treatment effectively excluded the aneurysm, but a type IIa endoleak with retrograde filling from the profunda femoris artery was identified during follow-up imaging. Despite attempts at endovascular embolization, the endoleak persisted, leading to a decision for open surgical intervention. The surgical procedure successfully resolved the endoleak and decompressed the aneurysm.

**Discussion:**

This case highlights the complexities of managing IIAAs and the potential for unusual collateral flows to complicate endovascular treatments, underscoring the importance of tailored surgical strategies and the need for close postoperative monitoring.

**Conclusion:**

This case illustrates the need for heightened awareness of the profunda femoris artery as a potential source of type IIa endoleaks in the context of internal iliac artery aneurysms.

## Highlights

• Isolated internal iliac artery aneurysm is an extremely rare entity.
• Most patients remain asymptomatic; however, rupture can lead to catastrophic outcomes.
• Retrograde filling from the profunda femoris artery is an unusual source of type IIa endoleak.
• The patient's risk factors and the characteristics of the aneurysm determine the most appropriate treatment approach.

## 1 Introduction

Internal iliac artery aneurysms (IIAAs) are a rare entity characterized by abnormal dilation of the internal iliac artery. Despite their infrequency, IIAAs present significant diagnostic and management challenges due to their potential for severe complications, including rupture and endoleaks. Endoleaks can compromise the effectiveness of endovascular repairs, resulting in continued blood flow into the aneurysm sac. Instances of retrograde filling from the profunda femoris artery have been documented in a few cases in the literature. This case highlights the possibility of the profunda femoris artery being a cause of type IIa endoleak.

This case report has been reported in line with SCARE guideline [12].

## 2 Case presentation

**Fig. 1:**
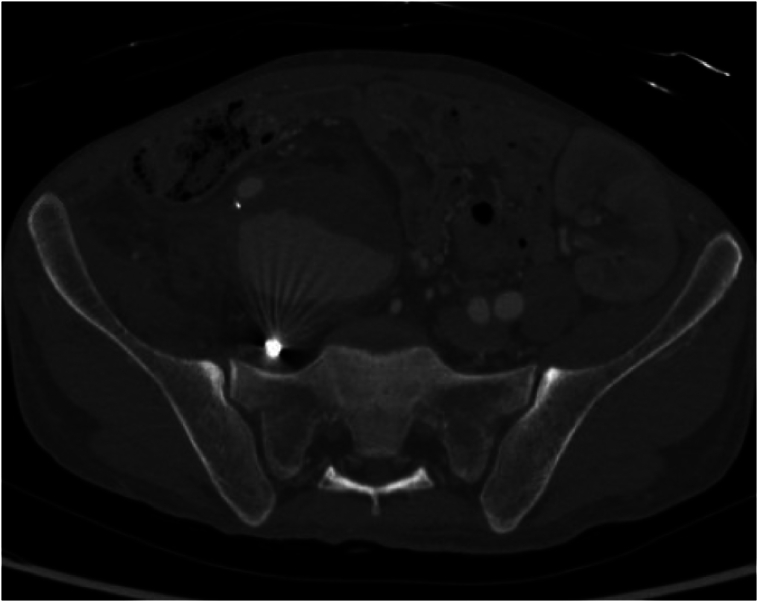
Large partially thrombosed right internal iliac artery aneurysm.

We present a case of a 32-year-old male with a known history of hypertension on oral 10 mg amlodipine once-daily and who has been diagnosed with end-stage renal disease and undergoing regular hemodialysis through Permacath. He has a history of two failed left-sided living-donor kidney transplants performed in 2018 and 2023. He was planned to have his first kidney transplant surgery based on the right external iliac artery. However, the transplantation was carried out on the left external iliac artery and vein for small caliper right vessels. During work-up for future transplant, a lower limb CT angiogram was done and revealed an incidental finding of a large partially thrombosed internal iliac artery aneurysm measuring 10 × 10 × 7.7 cm compressing the right common and external iliac veins as well as the external iliac artery with no signs of impending rupture (Fig. 1).

**Fig. 2:**
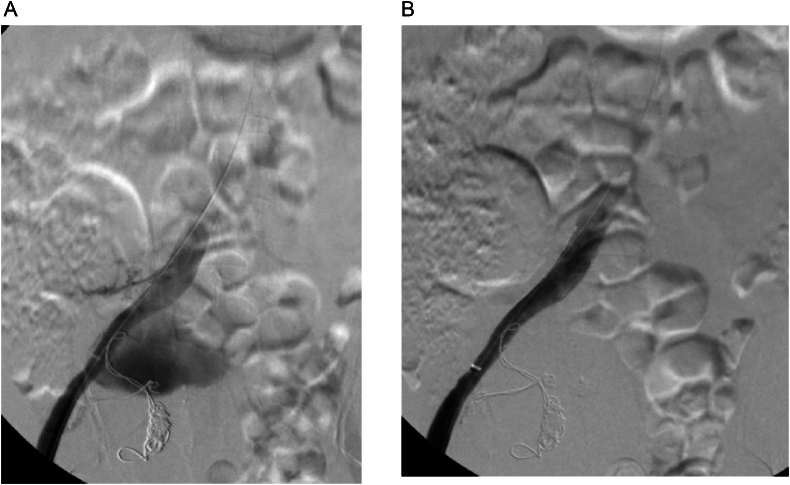
(A) Angiogram before stenting. (B) Completion angiogram revealed no flow to the aneurysmal sac.

**Fig. 3:**
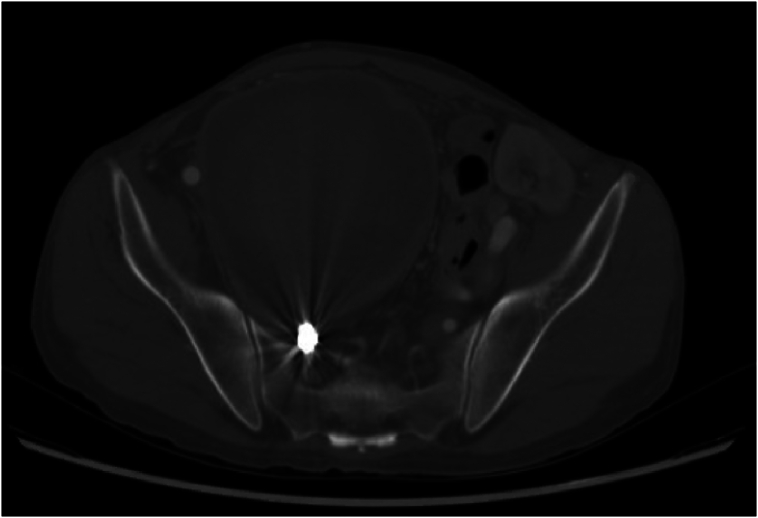
Increase in the size of the previously excluded right internal iliac artery aneurysm.

**Fig. 4:**
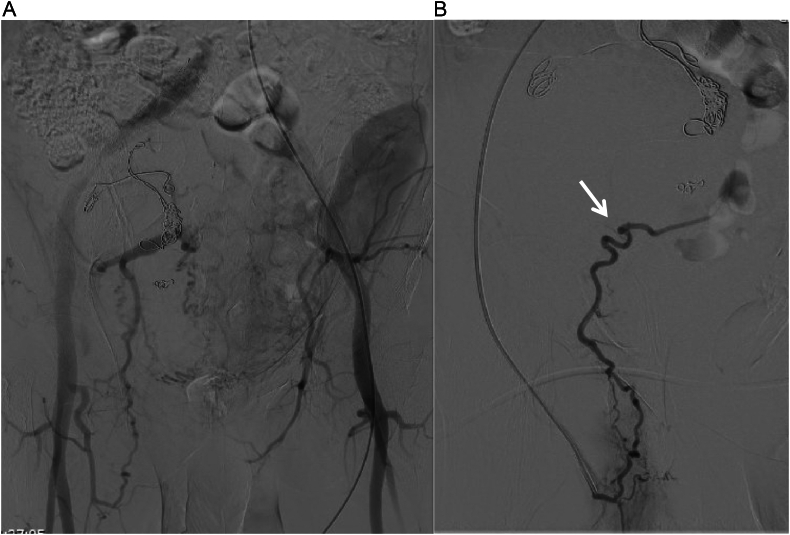
A and B: Type IIa endoleak via right profunda femoris artery (white arrow).

The patient was asymptomatic with an unremarkable abdominal and lower limb examination. Endovascular treatment was conducted utilizing coil embolization of the distal internal iliac artery followed by balloon expandable size (16 × 12 mm × 7 cm, Gore Viabahn VBX) extending from the common iliac to the external iliac artery, excluding the right hypogastric artery. The completion angiogram confirmed the absence of flow into the aneurysm with no signs of endoleak (Fig. 2A and B). Post-operatively, the patient was prescribed dual anti-platelets (once-daily oral Aspirin 81 mg and once-daily oral clopidogrel 75 mg) and was followed up in an outpatient setting. Eight weeks after the procedure, the patient was admitted to another local hospital due to upper gastrointestinal bleeding and a pulsatile abdominal mass in the right lower quadrant. A CT angiogram was conducted and showed an increase in the size of the previously excluded right internal artery aneurysm to 12.9 × 10 cm (Fig. 3). The stent was patent, with suspicion of type IIa endoleak and contrast retention in delayed images. Subsequently, the patient was taken for a conventional angiogram. It was observed that contrast was pooling within the aneurysm, with pelvic and thigh collaterals originating from the proximal right profunda femoris artery supplying the aneurysm (Fig. 4A and B), and therefore, the branch was embolized and despite that, type IIa endoleak persisted in the follow-up CTA. His case was discussed in a multidisciplinary meeting, and the decision was to proceed with open surgery to control the feeding source and to decompress the compressing aneurysm. He was electively brought to the operation room for exploratory laparotomy.

**Fig. 5:**
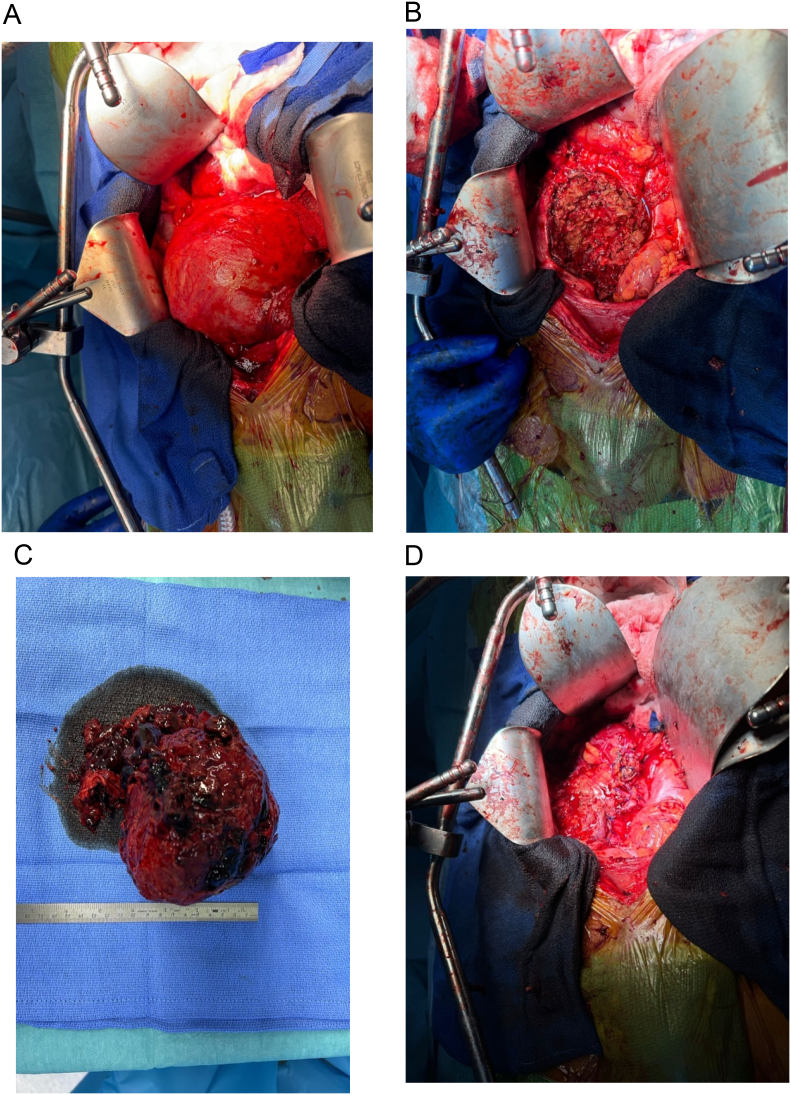
(A) A large internal iliac aneurysm occupying most of the patient's pelvic cavity. (B) Large thromus in the aneurysmal sac. (C) The thromus. (D) Closure of the aneurysmal sac.

Utilizing a midline incision, the abdominal wall was opened in layers, the peritoneum was opened, and a large size internal iliac aneurysm was found occupying most of the patient's pelvic cavity, compressing all surrounding structures. Dissection was carried around the aneurysm, and the aneurysmal sac was opened (Fig. 5A and B). A large thrombus was seen and removed, the feeding branch was suture ligated then the sac was closed (Fig. 5C and D).

The postoperative course was uneventful, and the patient was eventually discharged home on postoperative day 5. During follow-up in the outpatient clinic, he had no active complaint and was cleared for transplant from a vascular viewpoint.

## 3 Discussion

An isolated internal iliac artery aneurysm (IIAA) is defined as an abnormal dilatation of the internal iliac artery by more than 50 % of its normal diameter without concomitant aortic aneurysm. This condition is extremely rare, with an incidence rate of 0.03–0.4 % of the population [[1], [2], [3]]. Both iliac and aortic aneurysms share common etiologies and risk factors, including advanced age, hypertension, hyperlipidemia, and atherosclerosis, which are often exacerbated by lifestyle factors such as smoking and obesity. Despite their tendency to be degenerative, iliac aneurysms can also be caused by trauma, infection, connective tissue disorders, such as Marfan or Ehlers-Danlos syndromes, Behcet's disease, cystic medial necrosis, Takayasu's arteritis and fibromuscular dysplasia, iatrogenic post lumbar, pelvic, hip, or gynecologic surgeries [[4], [5], [6]].

The anatomical position of the internal iliac artery within the pelvis often leads to delayed diagnosis of IIAA, as many cases are asymptomatic until a rupture occurs, causing compressive symptoms or may be discovered incidentally during imaging studies—such as CT scans or ultrasounds—conducted for another pathology. McCready et al. highlighted that approximately 78 % of patients were asymptomatic, underscoring the challenges in early detection and the potential for catastrophic outcomes if the aneurysm ruptures [7].

In terms of management, a study by Laine et al. indicated that the risk of rupture significantly increases for internal iliac artery aneurysms measuring more than 4 cm in maximum diameter. This finding suggests that for smaller aneurysms, a conservative approach with vigilant monitoring may be appropriate, while elective intervention should be considered for aneurysms exceeding this critical size [8]. Surgical options include open surgical resection, which involves direct excision of the aneurysm, and endovascular techniques, such as coil embolization and endovascular exclusion using stent grafts and distal coil embolization. The endovascular approach has revolutionized the management of internal iliac artery aneurysms, offering a less invasive alternative to open surgical techniques. However, this minimally invasive strategy is not without complications. Complications arising from this approach include bleeding, rupture, and endoleak. Endoleak presents a unique challenge characterized by the persistent flow of blood into the aneurysm sac despite the deployment of an endovascular graft. This persistent flow can undermine the primary goal of the procedure, which is to ensure complete exclusion of the aneurysm and to prevent its expansion or potential rupture [9,10]. Endoleaks are classified into several types based on their etiologies and mechanisms, and they can arise from graft configuration, seal failures, or collateral circulation from nearby vessels [11].

Notably, exclusion of the internal iliac artery is generally well tolerated, largely due to the rich collateral circulation. Collateral vessels include the lumbar arteries, iliolumbar artery, middle sacral artery, and lateral sacral arteries. Interestingly, retrograde filling from the profunda femoris artery has been documented in a few cases in the literature [9,10]. In our case, the plan was endovascular exclusion of the right hypogastric artery. The endoleak was the reason for the operative approach, which eventually showed a feeding branch from the profunda femoris artery. Therefore, such causes of type IIa endoleak should be considered in similar cases.

Therefore, the patient's overall health, risk factors, the characteristics of the aneurysm should be taken into account to determine the most appropriate approach and achieve favorable outcomes.

## 4 Conclusion

Similar cases with rare feeding branches off the profunda femoral artery resulting in type II endoleaks can potentially be fatal as a result of aneurysmal expansion and rupture. Tailored approach, multidisciplinary team meeting and hybrid repairs are the key to positive outcomes.

## Author contribution

Abdullah G. Alsahwan: Principal investigator, designs, writing and review.

Ahmed Almumtin: Writing and review.

Mohammed Sadig: Literature review, and scientific data.

Osama Alahmadi: Literature review, and data collection.

Shagran Binkhamis: Overall supervision.

Omer Abdulrahim: Overall supervision.

## Consent

Written informed consent was obtained from the patient for publication of this case report and accompanying images. A copy of the written consent is available for review by the Editor-in-Chief of this journal on request.

## Ethical approval

King Faisal Specialist Hospital and Research Centre waived the need for IRB approval due to the absence of patient identification in the study's enrolled participant.

## Guarantor

Dr. Abdullah G. Alsahwan.

## Provenance and peer review

Not commissioned, externally peer-review.

## Research registration number

Not applicable.

## Funding

This study did not receive any funding.

## Conflict of interest statement

The authors have no competing interests.
